# Does gesture strengthen sensorimotor knowledge of objects? The case of the size-weight illusion

**DOI:** 10.1007/s00426-018-1128-y

**Published:** 2018-12-14

**Authors:** Wim Pouw, Stephanie I. Wassenburg, Autumn B. Hostetter, Bjorn B. de Koning, Fred Paas

**Affiliations:** 1 Department of Psychology, Education and Child Studies, Erasmus University Rotterdam, 3000 DR Rotterdam, The Netherlands; 2 Department of Psychological Sciences, University of Connecticut, Storrs, USA; 3 Human–Computer Interaction Institute, Carnegie Mellon University, Pittsburgh, USA; 4 Psychology Department, Kalamazoo College, Kalamazoo, USA; 5 School of Education/Early Start, University of Wollongong, Wollongong, Australia

## Abstract

Co-speech gestures have been proposed to strengthen sensorimotor knowledge related to objects’ weight and manipulability. This pre-registered study (https://www.osf.io/9uh6q/) was designed to explore how gestures affect memory for sensorimotor information through the application of the visual-haptic size-weight illusion (i.e., objects weigh the same, but are experienced as different in weight). With this paradigm, a discrepancy can be induced between participants’ conscious illusory perception of objects’ weight and their implicit sensorimotor knowledge (i.e., veridical motor coordination). Depending on whether gestures reflect and strengthen either of these types of knowledge, gestures may respectively decrease or increase the magnitude of the size-weight illusion. Participants (*N* = 159) practiced a problem-solving task with small and large objects that were designed to induce a size-weight illusion, and then explained the task with or without co-speech gesture or completed a control task. Afterwards, participants judged the heaviness of objects from memory and then while holding them. Confirmatory analyses revealed an inverted size-weight illusion based on heaviness judgments from memory and we found gesturing did not affect judgments. However, exploratory analyses showed reliable correlations between participants’ heaviness judgments from memory and (a) the number of gestures produced that simulated actions, and (b) the kinematics of the lifting phases of those gestures. These findings suggest that gestures emerge as sensorimotor imaginings that are governed by the agent’s conscious renderings about the actions they describe, rather than implicit motor routines.

## Introduction

Sensorimotor knowledge from the previous interactions with the environment plays an important role in planning and predicting everyday actions. For example, imagining object rotations is aided by hand gestures that simulate the manipulation of those objects (Chu & Kita, 2011; see also Alibali, Spencer, Knox, & Kita, 2011; Boncoddo, Dixon, & Kelly, 2010). It has been suggested that, by recruiting sensorimotor routines, remembered information of the previous interactions with objects becomes available that can support the predictability of the environment (Hostetter & Boncoddo, 2017; Pouw & Hostetter, 2016). Relevant to the present study, it has been found that gestures can also strengthen sensorimotor information in memory, which makes the information about object manipulation more prominent, and affects subsequent action on objects (e.g., Goldin-Meadow & Beilock, 2010). In the current study, we test this sensorimotor strengthening effect by exploring how co-speech hand gestures affect memory for sensorimotor information through the application of a well-known visual-haptic illusion known as the size-weight illusion.

The size-weight illusion (SWI) occurs when participants perceive a difference in the heaviness when lifting two differently sized objects that are, in fact, the same weight (i.e., haptic perception; for a review, see Buckingham, 2014). Specifically, a smaller object is experienced to be heavier than a larger object with the same weight. The exact mechanisms of the SWI are still under debate and may relate to the veridical perception of wieldability of an object (e.g., Amazeen & Turvey, 1996; Zhu & Bingham, 2011) and/or top–down expectations that small objects should be lighter than larger objects leading to expectation errors that bias experience (see Buckingham, 2014). The SWI is so robust that it persists even when participants have been told that the objects are the same weight and have been allowed to lift the objects with their eyes closed and feel the equivalent weight (Buckingham, 2014). The SWI is also present on the motor level, as it initially affects the gripping strength participants use to lift objects (Flanagan & Beltzner, 2000).

However, despite the persistence of the consciously experienced illusion, participants’ motor coordination eventually attunes to the lack of difference in weights after interacting with the objects multiple times. After lifting the objects 20 times, participants no longer overestimate the strength needed to lift a smaller object relative to a larger object (Flanagan & Beltzner, 2000). It appears that the perceptuo-motor system comes to attune to the veridical sensory feedback from lifting the objects, even while the conscious experience that the smaller object is heavier than the larger object remains unaltered. Therefore, after repeatedly (> 20 times) lifting the objects, there seems to be a disconnect in the sensorimotor knowledge available to the motor system as it plans a hand-grasp and the sensorimotor knowledge that is consciously available to the participant. This disassociation between implicit motor knowledge and explicit conscious knowledge has been explained by the different functional roles of the dorsal and ventral neural pathways (see Goodale & Milner, 1992). Importantly, because implicit motor knowledge and explicit knowledge diverge, the SWI is an interesting phenomenon for understanding how gestures might strengthen different kinds of sensorimotor knowledge.

Gestures differ from real actions, because they do not involve objects, and they differ from pantomimes, because they accompany speech. There is some evidence suggesting that gestures can affect how speakers think about the weight of the objects which they describe (Beilock & Goldin-Meadow, 2010; Cooperrider, Wakefield, & Goldin-Meadow, 2015; Trofatter, Kontra, Beilock, & Goldin-Meadow, 2015). Beilock and Goldin-Meadow (2010) had participants solve the Tower of Hanoi (TOH) task, in which participants manipulate discs of different sizes and corresponding weights according to specific rules. After solving the task, some participants were asked to explain how they solved the task and to use gestures as they did so. Then, all participants were asked to solve the task a second time in one of two conditions. In the No Switch condition, the second task was physically identical to the previous task. In the Switch condition, the discs’ weights were switched, so that the smallest disc was now the heaviest and could no longer be lifted with one hand. Beilock and Goldin-Meadow (2010) showed that the more participants gestured about lifting the smallest disc with one hand, the more impaired they were on the second version of the task when the weights had been switched. The basic effect has been replicated several times with similar methodological procedures and sample sizes (Cooperrider et al., 2015; Trofatter et al., 2015; but see Wassenburg, de Koning, & van der Schoot, 2018 for contrasting evidence), and the general explanation provided for it is that “using gesture to describe physical interactions with the environment generates strong mental representations that involve physical properties of the action and/or the environment (properties like weight)” (Trofatter et al., 2015, p. 8).

If this explanation is extended to gestures about objects that induce an SWI, participants’ memories for the weights of the objects should be affected by whether they gestured about lifting them. This could be manifest in either of two ways, depending on the nature of the sensorimotor knowledge that gestures strengthen (Flanagan & Beltzner, 2000). On one hand, gestures may bring the conscious perception of the objects’ weights more in line with the sensorimotor knowledge that is available to the motor system as participants actually lift the objects. For example, there is evidence that manual pantomimes (i.e., enactment of an interaction without a present object) approximate the kinematics of normal grasping actions to a high degree (Weiss, Jeannerod, Paulignan, & Freund, 2000) and that they simulate action-specific knowledge such as weight (Ansuini et al., 2016). Furthermore, some evidence suggests that gestures can help problem solvers to gain conscious awareness of their implicit *knowledge* (e.g., Church & Goldin-Meadow, 1986; Perry, Church, & Goldin-Meadow, 1988). Under this view, gesturing about lifting the objects repeatedly may align participants’ conscious perception of the weights of the objects more closely with the sensorimotor knowledge which they possess about how to actually lift them. This would result in a smaller SWI after gesturing about lifting the objects than after not gesturing.

On the other hand, it is also possible that gesturing about lifting the objects could strengthen the size of the illusion. Under this view, gesturing about lifting the objects could reflect and strengthen participants’ memory of the conscious perception that the smaller object is heavier, rather than their sensorimotor knowledge about how to actually lift the objects that are of the same weight. Pantomimes appear to be coordinated by a system concerned with perceptual aspects of objects rather than implicit action-specific (motor-relevant) properties of objects (Goodale, Jakobson, & Keillor, 1994). For example, when participants are asked to pantomime grasping a previously seen stick—that is designed to induce a visual illusion (i.e., Müller-Lyer illusion)—the hand aperture is biased by their illusory perception of the stick length. In contrast, when participants reach to grasp the actual sticks, the kinematics of their hand aperture do not reflect this illusory perception to similar degress and are, instead, more attuned to the actual lengths of the sticks (Westwood, Heath, & Roy, 2000). Thus, it is possible that gesturing about lifting the objects will further cement the illusory rendering that the smaller object is heavier in weight than the larger object.

In the present study, we test these possibilities by first having all participants practice solving a problem with pieces that induce a size-weight illusion. The problem involved physically moving the pieces 30 times, which is enough lifting experience with the pieces for the perceptuo-motor system to attune to the equal weights of the objects. Thus, at the end of the problem-solving task (see “Methods” for details), all participants were assumed to have accurate implicit sensorimotor knowledge that the weights of the pieces were equal while still experiencing an illusory conscious experience that the smaller object is heavier than the larger object (Flanagan & Beltzner, 2000). Then, participants were randomly assigned to one of three conditions. In the Control condition, participants performed a non-related task (i.e., solving a Sudoku puzzle). In the Gesture condition, participants were asked to explain the solution of the problem-solving task while gesturing. In the No-Gesture condition, participants were asked to sit on their hands while explaining the problem solution. It should be noted that, in gesture research, it is difficult to find a no-gesture explaining condition without confounding variables. On one hand, research has shown that prohibiting gestures can negatively affect the semantic richness of explanations (e.g., Hostetter, Alibali, & Kita, 2007). On the other hand, it is difficult to find participants who do not gesture spontaneously (e.g., Eielts et al., 2018) when explaining tasks involving spatial and motor skills, and such participants may have different spatial and motor skills than participants who do use co-speech gestures. In the present study, a prohibiting gesture condition was included to rule out an effect of explaining in and of it. Importantly, we did not expect semantic richness of verbal explanations to influence the SWI as the previous studies have shown no effects of gesture on speech content in this type of problem-solving task (e.g., Beilock & Goldin-Meadow, 2010). Similar exploratory analyses of speech content are included in the present paper to verify this (for results see Analysis 5 in “Appendix 2”). Finally, all participants provided two estimates of how heavy the task-relevant pieces felt using a magnitude estimation procedure commonly used to ascertain the magnitude of the SWI (e.g., Buckingham, Goodale, White, & Westwood, 2016). They were first asked to recall heaviness from memory (i.e., main variable of interest) and subsequently provided an estimate while holding the objects (i.e., this more direct estimate of the SWI serves as a materials check). Participants who experience the SWI should report the small cube as being heavier than the large cube in both measures. Furthermore, we predicted that the magnitude of the experienced SWI (i.e., recalled heaviness) might depend on whether participants had gestured about the task. Compared to participants who have not gestured about lifting the objects, participants who have gestured about lifting the objects should experience either a larger SWI (if gestures strengthen the consciously perceived illusion that the smaller object is heavier) or a smaller SWI (if gestures allow access to implicit sensorimotor knowledge about actual lifting).

In addition to these pre-registered hypotheses (https://www.osf.io/9uh6q/), we conducted a series of exploratory analyses pertaining to how gestures are related to specific aspects of sensorimotor knowledge. In a recent conceptual replication of the original TOH studies, researchers failed to obtain the original sensorimotor strengthening effect (Wassenburg et al., 2018). However, they did find that task-relevant gestures (i.e., movements from left-to-right) were related to relatively slower task performance in the switched condition (solving TOH from right to left), similar to the correlation between amount of one-handed movements and task solution reported by Beilock and Goldin-Meadow (2010) in the Switch condition. These results may be explained by the hypothesis that gestures reflect (rather than affect) the way that participants think about the task. In the present study, we addressed this hypothesis by examining how gesture form is related to the magnitude of either the recalled or the perceived illusion. We examined how often gestures were produced that closely mirrored the lifting motion involved in the actual task (e.g., lifting with two hands, rather than one), to see if producing such congruent gestures would be associated with a larger or smaller illusion. We also used a Frame Differencing Method (FDM; Brookshire, Lu, Nusbaum, Goldin-Meadow, & Cassasanto, 2017; Romero et al., 2017; Pouw et al., 2018) to measure the velocity of two-handed lifting gestures to explore whether participants who report that the objects are heavier would move their hands more slowly as they gestured about lifting them. If gestures reflect such sensorimotor knowledge in their kinematics, this would provide strong evidence that gestures are based in sensorimotor know how.

## Method

### Participants

This study was conducted in accordance with the guidelines of the ethical committee of the Department of Psychology at Erasmus University Rotterdam. As stated in the pre-registration (https://www.osf.io/9uh6q/), participants (*N* = 162, *N* − exclusions = 159) were recruited from a Dutch University for course credit or a small monetary reward. All participants provided informed consent prior to their inclusion in the study. The sample was largely female (73.6%), right-handed (86.7%), and had a mean age of 20.78 years (SD = 2.79, range 18–38). Gender (*χ*^2^ = 2.06, *p* = .357) and hand dominance (*χ*^2^ = 5.82, *p* = .444) ratios were equal across conditions. The sample size was based on G*Power calculations for a between-subjects design with three groups: a medium-effect size (Cohen’s *F* = 0.25), a power of 80%, and an alpha of 5% (see Appendix C of the pre-registration for G*Power calculation specifications). Note that three participants were excluded from the data set. One participant did not understand the instructions and technical problems resulted in the loss of video data from two other participants. As stated in the pre-registration, data collection was continued until there were 53 participants per condition.

### Materials

**Fig. 1:**
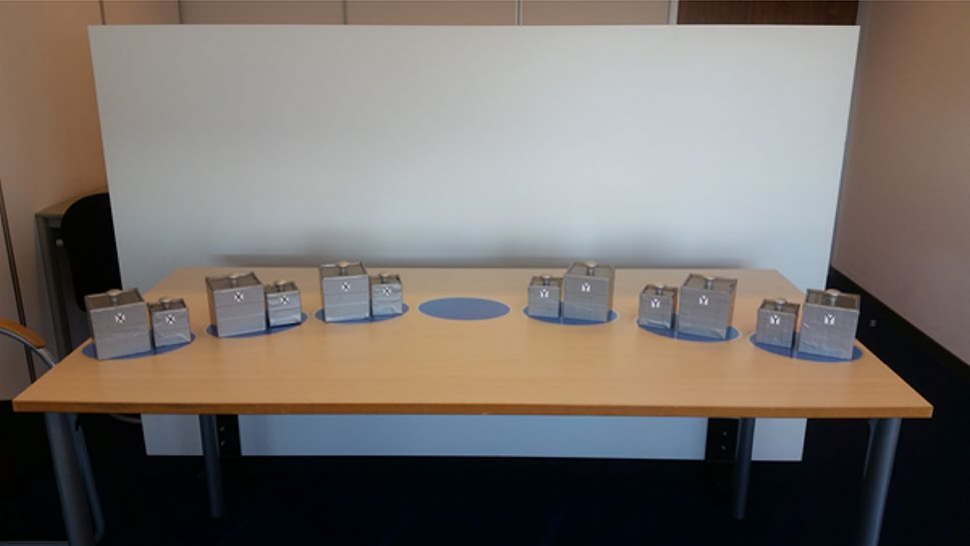
Cube pairs (six identical sets). (Color figure online)

**Fig. 2:**
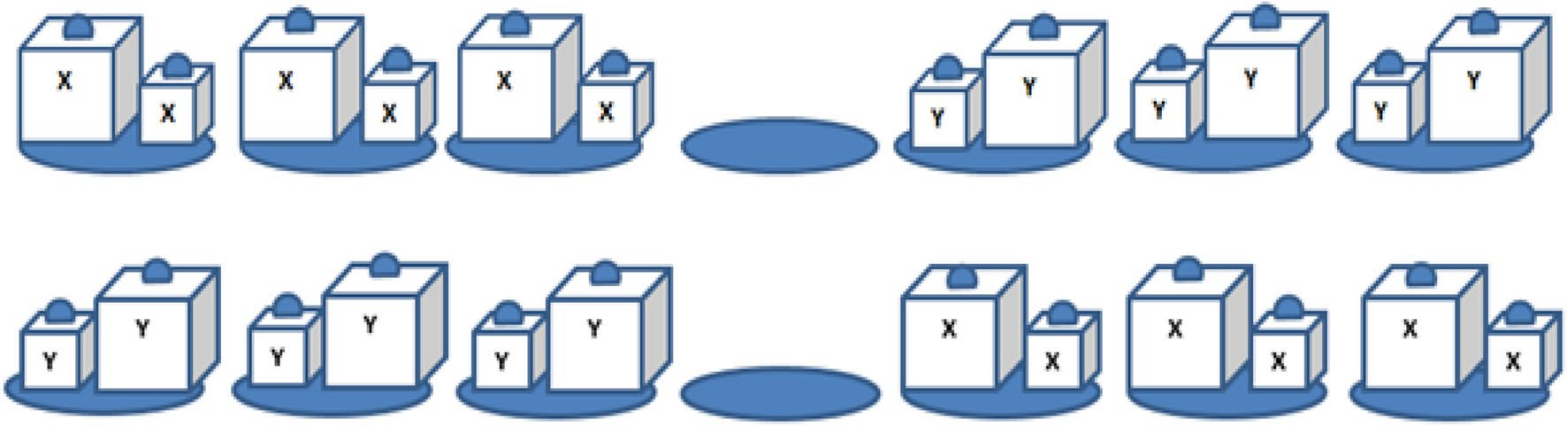
Schematic representation of the task set-up of the adapted Frog Leap task. Upper picture shows begin state; lower picture shows the target state. (Color figure online)

*Problem-solving task* We used a physical version of the Frog Leap computer task (e.g., van Gog, 2011), which requires the transformation of a begin state into a goal state given specific rules. In our version of the task, participants moved pairs of cubes that each consisted of one large and one small cube (see Fig. 1). The goal is to move the pairs on the right side to the left, and the pairs on the left side to the right, by lifting both cubes in a pair simultaneously to “jump” them over another pair. A pair of cubes could only be placed on an empty designated spot (indicated by blue laminated ovals). Participants moved one pair at a time by lifting both cubes in the pair by their handles. Pairs could be moved only one step forward and backward moves were not permitted. A pair from one side was allowed to jump over one pair from the opposite side (and vice versa). Participants transformed the task from begin state to end state (see Fig. 2), and again from end state to begin state, which took 30 moves to complete.

*Cubes* Participants were presented with six identical pairs of cubes (see Figs. 1, 2). Each cube pair consisted of a small (7 cm × 7 cm × 7 cm) and a large cube (10 cm × 10 cm × 10 cm) that had been filled with fine-grained sand to weigh exactly 450 g. This resulted in a density ratio between the small and large cubes of approximately 3:1 (i.e., 1.31 g/cm^3^ for small cubes and 0.45 g/cm^3^ for large cubes). The previous research has shown that although equal in weight, a difference of 2.1 cm^3^ in size will result in a reliable size-weight illusion; a smaller cube of 7.4 cm^3^ was experienced to be about 20% heavier than the equally heavy but larger cube of 9.3 cm^3^ (i.e., with a density ratio of 2:1; Buckingham et al., 2016). All cubes were wrapped in silver–gray duct tape and marked with either two “x” or two “y” symbols to distinguish whether it began on the left side or right side. A handle was affixed to the top of each cube.

*Camera* All participants were tested in a lab with a built-in video camera at the approximate eye height of a seated person. Participants were seated directly in front (i.e., 0° angle) of the camera at a distance of 280 cm. Care was taken to keep the seating position relative to the camera identical across participants to allow for exploratory analyses on the kinematics of gesture (e.g., Hilliard & Cook, 2017).

*Heaviness ratings* Heaviness of the small cube and the large cube was measured on a ten-point scale running from 1 = ‘very light object’ to 10 = ‘very heavy object’ using the question “How heavy did you perceive the [small or large] cube to be?”. A visual representation of the heaviness scale was presented, and participants provided verbal responses. The difference between the two heaviness ratings for large and small cubes indicates the magnitude of the size-weight illusion (i.e., heaviness small cube > heaviness large cube). This is a common method to measure the size-weight illusion when a single heaviness rating per cube is used (e.g., Buckingham et al., 2016). Note that the order of heaviness ratings for the small and large cubes was counterbalanced.

### Design

The study followed a 2 × 2 × 3 mixed design with size (small cube vs. large cube) as a within-subjects factor, and order (small–large vs. large–small order of heaviness ratings) and condition (Gesture Condition vs. No-Gesture Condition vs. Control Condition) as between-subjects factors. Participants were randomly assigned to one of three conditions. Participants who were asked to explain the task were either encouraged to use their hands (Gesture Condition) or were prohibited from using their hands (No-Gesture Condition). Participants who did not explain the task solved a Sudoku puzzle instead (Control Condition). The main outcome variable was Recalled Heaviness (from memory). An additional outcome variable, Perceived Heaviness, was added to check whether the materials were appropriate to induce a size-weight illusion.^1^

### Procedure

Participants were instructed that they would learn to solve a game puzzle. First, participants were given an instruction sheet containing the rules and goal of the game (“Appendix 1” of the pre-registration). After indicating that they understood the rules, they completed a scripted problem-solving routine of the task (“Appendix 2” of the pre-registration; practice phase), containing 15 steps to solve the puzzle once and 15 steps to solve the puzzle another time, but in the opposite direction (i.e., the end state was the begin state and vice versa). This way, the positions of cubes were perfectly counterbalanced within participants (i.e., all participants lifted both small and large cubes with both left and right hands and moved them to the left vs. right an equal number of times). Solving the puzzle twice also ensured that all participants used 30 lifting trials as they practiced the puzzle. We required 30 lifting trials to achieve the state demonstrated in the previous research where participants no longer produce higher gripping forces for the smaller cube (i.e., the unconscious motor knowledge has attuned to actual weight of the cubes), but, nevertheless, still experience a conscious size-weight illusion (Flanagan & Beltzner, 2000).

Participants were instructed that there was no time constraint and that it was important that they worked as accurately as possible. If the participant did make a mistake, the experimenter intervened by placing the misplaced pieces back and performing the right step instead. Only 12 participants made one or two mistakes (control condition *n* = 4, gesture condition *n* = 3, and no-gesture condition *n* = 5). Importantly, the experimenter did not pick up the pieces, but moved the pieces by sliding the placeholders. This ensured that participants did not derive weight information from observing actions of the experimenter. The total time spent on the task was noted by the experimenter.

After the practice phase, the puzzle task was removed from the participant’s sight for the rest of the experiment. In the control condition, participants solved a Sudoku puzzle for 2 min. In the explanation conditions, the table was moved away to ensure that participants had a full motion range fully visible to the camera. Participants were asked to explain the 15 solution steps of the task, as though talking to someone who was familiar with the rules, but not the solution of the puzzle. To equate the amount of time spent explaining with the time spent in the control task, participants were instructed to stop explaining after 2 min, though this time requirement time was not mentioned to participants before their explanation. Half of the participants in the explanation conditions were instructed to use their hands while explaining the steps (gesture condition), whereas the other half were asked to put their hands under their legs to prevent them from using them (no-gesture condition). In line with the previous gesturing studies (e.g., Beilock & Goldin-Meadow, 2010), we explicitly asked participants to (not) use their hands. In both conditions, if the participants were silent for 10 s or if they lost track of the imagined solution steps, they were instructed to try again and start with the first step. These prompts were repeated if necessary to ensure that the full 2 min were used for active explanation.

After 2 min of explaining or solving the Sudoku, participants were given a visual representation of the ten-point heaviness rating scale and asked to provide a verbal rating of the recalled heaviness of the small and large cubes that they had lifted in the task (order was counterbalanced across participants). Participants were instructed that there were no wrong or right answers and to provide the first answer that came to mind. The majority of participants (*N* = 138) were then asked to lift the cube pair in the same way as they did during the task (i.e., using precision grip) and to report the perceived heaviness of each cube, while both were held. The reported ratings were recorded by the experimenter.

### Coding

**Fig. 3:**
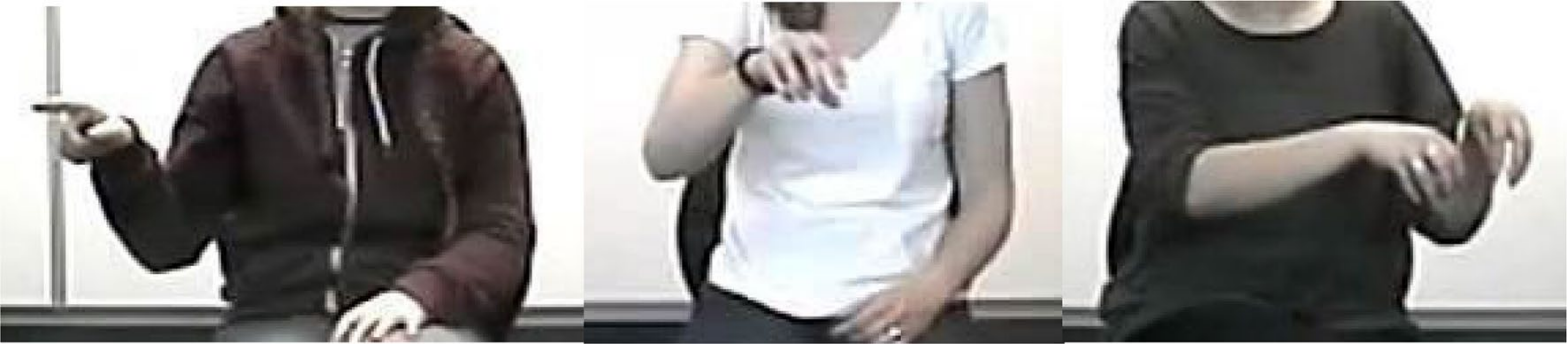
Example of deictic, one-handed, and two-handed grasp/move gestures

For both explanation conditions (i.e., gesture and no-gesture condition), speech was transcribed for subsequent analysis. In the no-gesture condition, video data were rechecked for gestures, and none were observed. All gestures in the gesture condition were coded in the context of concurrent speech, and three categories of gestures were counted (for examples, see Fig. 3): (1) deictic gestures (i.e., pointing to an object or location), (2) gestures representing grasp or move actions with one hand, and (3) gestures representing grasp or move actions with two hands. Note that, in the actual task, pairs of blocks were lifted simultaneously with one block in each hand. As such, gestures representing grasp or move actions with two hands most closely resemble the actions used in the problem task. All 53 videos were coded by one coder (one author of this paper) whose scores were used in analyses. Because coding was a time-intensive task, only a subset of the data (18%) was coded by a second coder (and author of this paper) to establish reliability. The reliability of the subset of participants may be generalized to the full sample (Hallgren, 2012) and this approach is in line with the previous studies using gesture coding (e.g., Chu et al., 2014; Cook & Goldin-Meadow, 2006). To quantify the agreement between the two coders, Krippendorff’s alpha (inter-rater reliability for two coders of a ratio variable) was calculated for the gesture count of each category separately (using the SPSS macro of Hayes & Krippendorff, 2007). All alphas were above 0.96.

### Exploratory: gesture kinematics using frame differencing method

We obtained the rate of movement (velocity) of gestures using a Frame Differencing Method (FDM; current sampling rate 25 frames per second). FDM utilizes an algorithm that computes the number of pixels that change from frame to frame from a video recording (using Python code made publicly available by Brookshire et al., 2017). This method provides an indication of gross movement through time—and is reliable compared to the other methods such as Polhemus or Kinect (see Romero et al., 2017)—which can be used as an estimate of velocity of hand-gesture movements. We were interested in the velocity of lifting gestures, specifically, to see if the kinematics of such gestures during the lifting phase is related to participants’ estimated heaviness of the cubes. We (1) computed velocity traces for each participant’s video data using the FDM method, (2) z-normalized the velocity traces for each participant, such that individual differences in body size (and thus pixel change/velocity) are rescaled, and (3) applied a Butterworth low-pass filter (10 Hz) which smooths noise-related fluctuations (for data manipulation procedures, see R script on OSF: https://www.osf.io/9uh6q/).

Subsequently, an independent rater who was unaware of the weight judgments given by the participants used the annotation software ELAN (Lausberg & Sloetjes, 2009) to identify each two-handed grasping gesture that simulated a lifting movement of the objects. Two-handed gestures that did not include a lifting movement were not used for this analysis.^2^ Using custom-made script in R, we merged the ELAN gesture codings with the velocity trace data. We then used this to identify the velocity trace of the first 500 ms of each lifting gesture that was produced by each participant. We focused on this time frame, because physics dictates that, everything else being equal, heavy objects will have more inertia (are more resistant to change in motion) as compared to lighter objects. Thus, if a gesture simulates the inertial forces of lifting a heavy object, the velocities generated during the first 500 ms of the lifting gesture will be lower (i.e., “the movement will be slower”) as compared to gestures simulating manipulations with lighter objects with low inertia. Indeed, velocity or positive peaks in velocity have been used to quantify language-induced weight-expectancy effects for actual lifting movements (e.g., Scorolli, Borghi, & Glenberg, 2009). Furthermore, (average) velocity for lifting phases (rather than the reach and placing phases), is one of the defining perceptual cues that determine whether observers can see whether a light vs. heavy object is being lifted (Alaerts, Breukelaar, Swinnen, & Wenderoth, 2012). Thus, we focused on velocity for the initial lifting phase (500 ms) of the lifting gesture.

We also computed the combined heaviness rating of the objects for each participant, which is the average of the recalled heaviness rating for the small and large objects. Because we are interested in how weight judgments affect gesture kinematics, we computed the average gesture velocity trajectory for all participants who reported a particular heaviness rating. Thus, if six participants judged objects to have a combined weight of 3, the average velocity trajectory (500 ms trajectories) was calculated for the gestures of those participants. Thus, for each observed heaviness rating, we have a stereotypical (i.e., averaged) gesture velocity trajectory. This way, we can compare how gesture velocity trajectories differ as a function of whether they were produced by someone with lower or higher weight judgments.

## Results

The method and results of this study were pre-registered. We, first, present the results pertaining to hypotheses and analyses that were pre-registered. As described, these confirmatory analyses did not support the predictions stated in the pre-registration. We, thus, conducted several further analyses to help contextualize and understand these null findings. Such analyses are labeled as exploratory in the sections that follow.

### Descriptive statistics

In Table 1 in “Appendix 1”, the means, standard deviations, and correlations are provided for the main variables in this study.

### Planned confirmatory analysis: effect of condition on recalled heaviness ratings

**Fig. 4:**
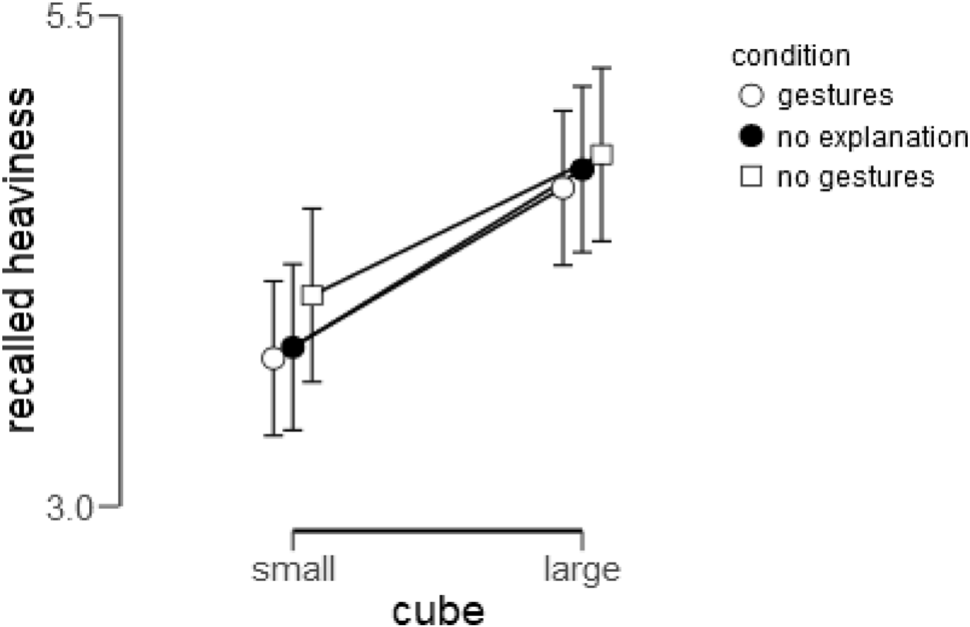
Effect of condition on recalled heaviness. Error bars indicate 95% confidence intervals

As stated in the pre-registration, we performed a 2 × 3 mixed ANOVA (including interactions), with Size (small vs. large cube) as a within-subjects factor, and Condition (gesture, no-gesture, control) as a between-subjects factor. Note that counterbalanced conditions for the order of heaviness ratings (small–large or large–small) did not affect overall heaviness ratings, *F*(1, 157) = 0.07, *p* = 0.799, or differences in heaviness ratings, *F*(1, 157) = 0.036, *p* = 0.849, and will, therefore, not be adopted in the tested models (as planned in the pre-registration). Surprisingly, in contrast to the typically reported size-weight illusion (SWI), participants recalled the large cube as being heavier (*M* = 4.71, SD = 1.83) than the small cube (*M* = 3.88, SD = 1.82), Size: *F*(1, 156) = 23.63, *p* < .001, *η*_p_^2^ = 0.13, *d* = − 0.39. This indicates that there was an inverted SWI when participants reported the cubes’ heaviness from memory. As can be seen in Fig. 4, there was no statistically significant effect of Condition on recalled heaviness, Condition: *F*(2, 156) = 0.47, *p* = .627, *η*_p_^2^ = 0.006. Finally, the difference in heaviness ratings for small vs. large cubes did not differ as a function of Condition, Size × Condition: *F*(2, 156) = 0.11, *p* = .892, *η*_p_^2^ = 0.001. This indicates that the SWI was unaffected by whether participants explained or gestured about the task.

### Exploratory analysis 1: evidential value for null findings

Given the null results for our confirmatory analyses with regards to condition, we performed an additional exploratory Bayesian analysis to estimate the likelihood of the observed data given the null hypothesis. All Bayes’ factors (BF) reported in this manuscript were computed by JASP (JASP Team 2016, Version 0.8.4), which operates with the default priors *p*(*M*) = 0.5 (Cauchy prior of *h* = 0.75; Rouder, Morey, Verhagen, Swagman, & Wagenmakers, 2017). Jeffreys (1961) classifies the strength of effects with respect to Bayes’ factors (BF)^3^ as follows: no evidence BF = 1, anecdotal evidence BF = 1–3, substantial evidence BF = 3–10, strong BF = 10–30, very strong BF = 30–100, and decisive BF > 100.

We performed a Bayesian 2 × 3 mixed-design ANOVA (including interactions), with Size (small vs. large cube) as within-subjects factor and Condition as between-subjects factor. The Bayesian analysis for the between-subject effect of Condition yielded BF_01_ = 12.13 (strong evidence). This suggests that the null hypothesis, predicting no differences in heaviness ratings as a function of Condition, is 12.13 times more likely given the data as compared to a model predicting differences between groups. The interaction effect of Condition and Size yielded decisive evidence for the null hypothesis (BF_01_ = 191.09). Note that Bayesian analyses also provided decisive evidence against the null hypothesis for the within-subjects effect of Size, where we obtained an inverted SWI (BF_10_ > 1000). In summary, we obtained strong to decisive evidence that Condition did not affect heaviness ratings of the small cube vs. large cube when made from memory.

### Exploratory analyses of perceived heaviness ratings

**Fig. 5:**
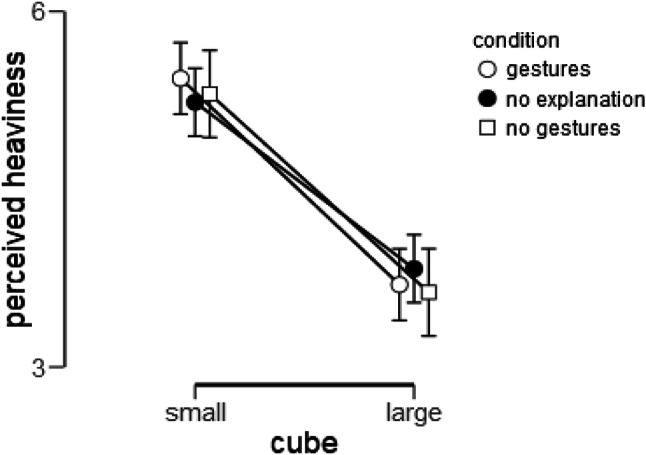
Effect of condition on perceived heaviness. Error bars indicate 95% confidence intervals

*Analysis 2a: materials check* We did not obtain the typical SWI when participants recalled heaviness of the cubes from memory. In fact, participants rated the large cube as heavier than the small cube when rating heaviness from memory of their previous experience (an inverted SWI). However, when heaviness was rated while actually holding the objects at the end of the experiment (i.e., as a direct estimate of the SWI), the typical SWI appeared as expected, indicating that the cubes did, indeed, generate a reliable SWI in this sample. As can be seen in Fig. 5, the small cube (*M* = 5.32, SD = 1.61) was perceived as heavier than the large cube (*M* = 3.72, SD = 1.37), Size: *F*(1, 138) = 153.70, *p* < .001, BF_10_ > 1000, *d* = 1.05.

*Analysis 2b: effects of condition* We further assessed whether Condition affected perceived heaviness, while the cubes were being held. We performed a similar 2 × 3 mixed-design ANOVA (including interactions), with Size (small vs. large cube) as a within-subjects factor and Condition as a between-subjects factor. There was no statistically significant effect of Condition, *F*(2, 156) = 0.07, *p* = .934, BF_01_ = 10.06, *η*_p_^2^ = 0.001, or Condition × Size interaction, *F*(2, 136) = 0.63, *p* = .535, BF_01_ = 58.92, *η*_p_^2^ = 0.009, on perceived heaviness ratings.

### Exploratory analyses of possible covariates

**Fig. 6:**
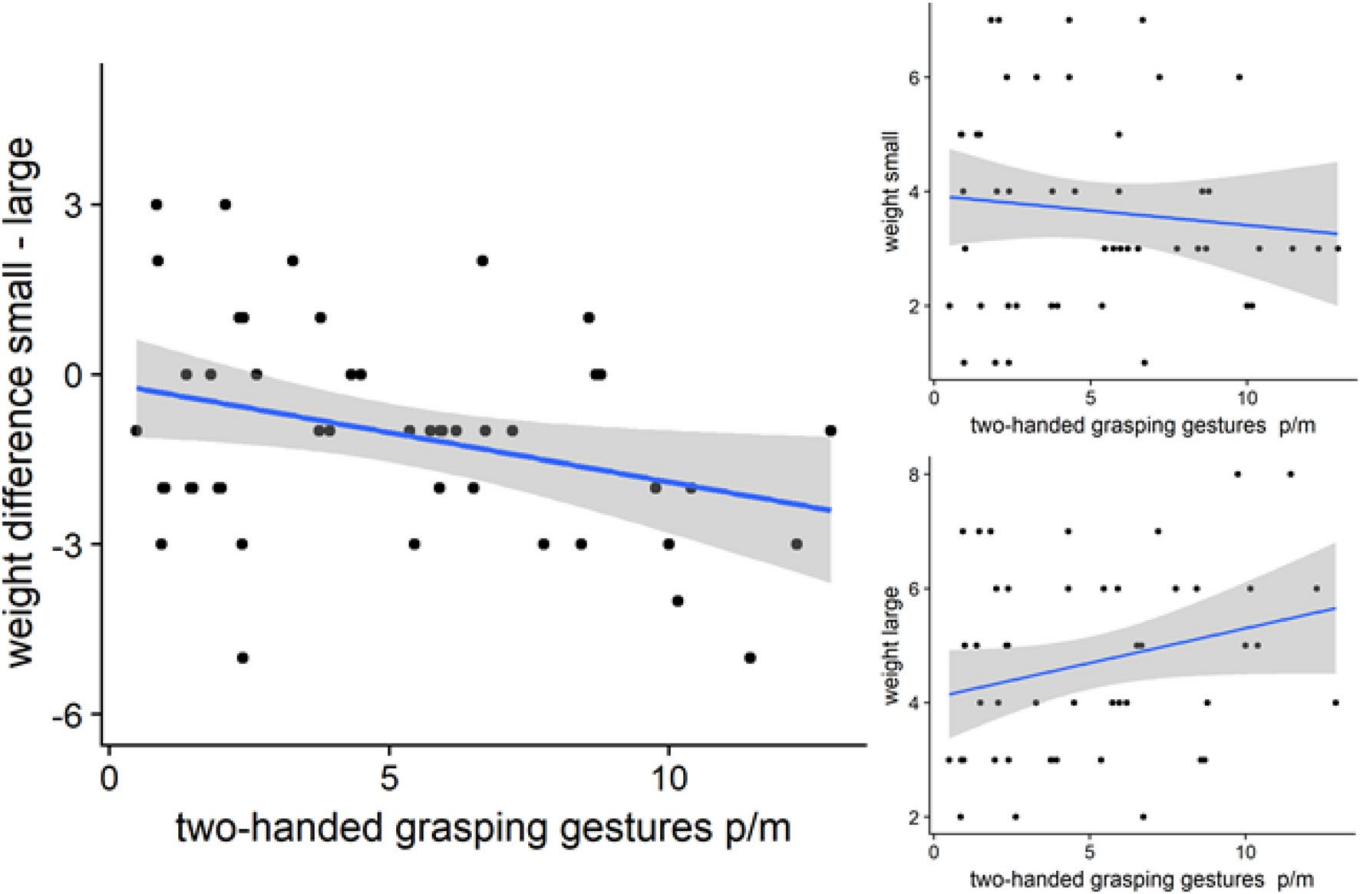
Two-handed gestures and heaviness of the objects, as rated from memory. Regression slopes and confidence intervals for the relation between two-handed grasp/move gestures and recalled heaviness ratings for the (differences of) small cube (upper right) and large cube (lower right). Lower values on the difference scores (left graph) indicate that the larger cube is rated from memory as heavier than the smaller cube, leading to negative scores. Note that some participants (those with positive difference scores) did recall the smaller cube as heavier than the larger cube, indicating memory of the consciously experienced illusion. Figures were generated with statistical software R (R Core Team, 2017). (Color figure online)

*Analysis 3: individual differences in gesture* The previous research has shown that the number of task-relevant gestures mediated magnitude of the effects on problem-solving performance (Beilock & Goldin-Meadow, 2010; Wassenburg et al., 2018). Table 1 (“Appendix 1”) provides a correlation matrix of the number of observed gesture types that occurred per minute (deictic, one-handed grasp/move, and two-handed grasp/move) and heaviness ratings of the cubes. In addition, for the gesture condition, the mean gesture rate per minute is provided in this table. The most striking result in this correlational analysis was that more two-handed grasp/move gestures were highly correlated with a smaller difference in recalled heaviness of the two cubes, *β* = − 0.37, *t*(52) = − 2.82, *p* = .007, BF_10_ = 6.54. As can be seen in Fig. 6, the difference was primarily carried by judgments that the larger cube was heavier, *r* = .29, *t*(52) = 2.18, *p* = .034, BF_10_ = 2.11, while the numerical judgments for the recalled heaviness of the smaller cube were unrelated to the number of two-handed gestures per minute; *r* = − .15, *t*(52) = − 1.08, *p* = .284, BF_10_ = 1.90. Note from Table 1 that there were no significant correlations between other types of gestures (deictic and one-handed grasp/move gestures) and difference in heaviness recalled from memory. The significant negative correlation, *r* = − .28, *t*(52) = − 2.11, *p* = .040, BF_10_ = 1.67, between the total number of gestures (sum of deictic, one-handed and two-handed grasp/move gestures, and all other undefined gestures) and difference in heaviness recalled from memory thus seems to be carried by the number of two-handed grasp/move gestures. Also note that there were no significant correlations of gesture with respect to perceived heaviness, while the objects were in hand.

*Analyses 4 and 5: other individual differences* In Analyses 4 and 5, we explored individual differences in practice time, explanation time, and speech content, to determine whether these variables may have affected our results. Results showed that neither practice time nor explanation time was statistically significant when added as covariates to the confirmatory analyses. Furthermore, speech rate and speech content could not account for the effect of two-handed gestures on recalled heaviness. For a more detailed description of the results, see “Appendix 2”.

**Fig. 7:**
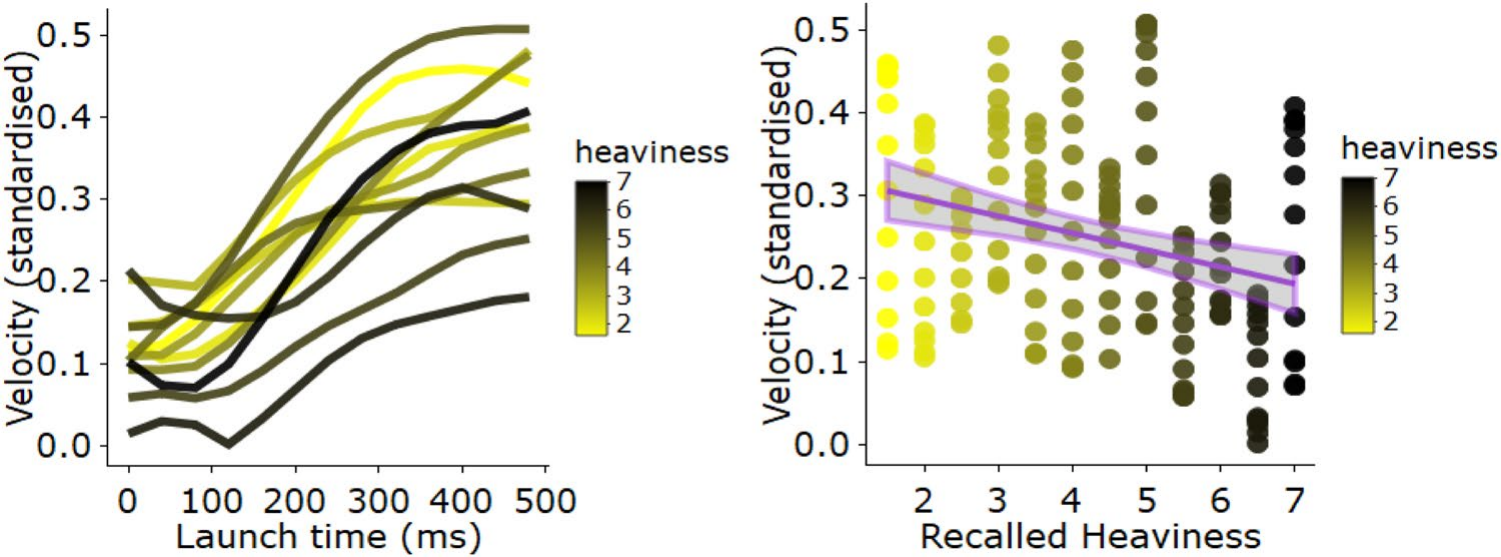
Velocity traces for the two-handed gesture launches and scatter plot for velocity samples as related to heaviness ratings. Left panel: mean velocity traces (500 ms) for each recalled heaviness rating (blackened lines indicated higher weight estimates; more yellow/lighter lines indicate lighter recalled heaviness ratings). A steeper positive slope of the velocity trajectory indicates that a gesture is moving more quickly; that is, velocity of the movement is increased in a shorter amount of time (i.e., higher acceleration) as compared to a more negatively sloped trajectory. Right panel: scatter plot with regression slope and SE interval (heaviness represented in color to match left panel) and *x*-axis. (Color figure online)

*Analysis 6: exploratory analyses of gesture kinematics* Figure 7 shows the velocity traces for gesture launch phases (500 ms) averaged for participants and combined heaviness rating from memory (min = 2, *M* = 4.24, max = 7). Participants who rated objects as heavier (darker black lines) produced gesture launches with lower velocity and acceleration than participants who rated objects as lighter (lines with more yellow coloring), as indicated by less steep trajectories and earlier velocity stabilization. This suggests that participants who recalled objects as heavier gestured about the objects as though they would be more difficult to lift. A correlation analyses confirmed that the higher the recalled heaviness of the small cube and large cube (combined), the lower the velocity values, *r*(154) = − 0.29 (~ medium-effect size), *p* < .001, BF_10_ = 78.45 (see Fig. 7 right panel for the relation of heaviness and velocity data). Note that this relationship between velocity and weight estimates was not present for weight estimates that were made when objects were held, *r*(154) = 0.04, *p* = .52, BF_01_ = 6.41.

## Discussion

The present pre-registered study assessed how gestures support memory for sensorimotor information of the weight of task-relevant objects during problem solving. The pieces were designed to induce the visual-haptic size-weight illusion (SWI), which is a consciously experienced perception that smaller objects are heavier than larger objects when the two are actually the same weight. Because gestures have been suggested to affect sensorimotor knowledge (Beilock & Goldin-Meadow, 2010; Cooperrider et al., 2015; Goldin-Meadow & Beilock, 2010; Trofatter et al., 2015), it was predicted that the magnitude of the SWI would be affected when participants gestured about the task-relevant objects. On one hand, gestures could increase the size of the illusion, suggesting that gestures strengthen, in memory, the consciously perceived illusion that the pieces differ in weight. On the other hand, gestures could decrease the size of the illusion, suggesting that gestures strengthen the implicit sensorimotor knowledge that the pieces have the same weight. We found no evidence to support the prediction that gesturing about the problem pieces affected the weight estimates of the pieces in either direction. Instead, our results show that judgments about the weight of the cubes were unaffected by whether participants gestured.

Interestingly, our exploratory analyses revealed that speakers who produced the most gestures mimicking the form of the two-handed lifting and moving actions involved in the task were also the most likely to experience a large inverted SWI in their memory for the pieces. Namely, the number of two-handed grasp gestures produced per minute by participants who were told to gesture as they explained the task was reliably associated with their memory of how heavy the pieces were. This finding, thus, replicates the previous reports that action-relevant gestures are reflective of sensorimotor cognition (e.g., Beilock & Goldin-Meadow, 2010; Wassenburg et al., 2018). Furthermore, we provide an additional evidence that the velocity with which speakers lifted their hands as they gestured is related to their memory of the objects’ weight. When speakers thought of the objects as heavy in their memory, they gestured about them as though they would be more difficult to lift, as indicated by lower average velocity traces. This provides evidence that speakers embody weight information in the kinematics of their co-speech gestures (as predicted by Mangelsdorf, Cooperrider, & Goldin-Meadow, 2017; see also Ansuini et al., 2016 for related findings with pantomimes; see also Cook & Tanenhaus, 2009, for how gesture kinematics is affected by the previous task experiences). Future research could specifically focus on how the kinematics of gestures about interactions with objects might be similar to and different from the kinematics of actually interacting with the objects. Indeed, although gestures might approximate object-oriented actions in some respects (as shown here), they are very likely to diverge on the other aspects wherein kinematics are constrained by dynamics that arise by actual wielding of objects (e.g., Runeson & Frykholm, 1983).

Furthermore, this is the first evidence that gestures reflect sensorimotor information that is consciously perceived, rather than the precise kinematics of a previous action. Participants who believed the objects were heavier gestured about them with slower lifting velocities than participants who believed that they were lighter, even though all objects in the present study were of exactly the same weight and required identical lifting velocities as participants solved the task. This is interesting, because it suggests that gestures are not direct recreations of the previous actions; rather, they are actions that are filtered through the participants’ conscious beliefs about those actions. This finding aligns with claims that gestures are representational (Novack & Goldin-Meadow, 2017) or simulated (Hostetter & Alibali, 2008) actions. Thus, actions and gestures are critically different, because gestures cannot and do not attune to action-relevant information in the environment the way that actual actions on objects do (Laimgruber, Goldenberg, & Hermsdörfer, 2005; Kuntz, Karl, Doan, & Whishaw, 2018; Runeson & Frykholm, 1983), at least not when the objects are not present (cf. Chu & Kita, 2016). Although gestures may have their origin in the action system, their execution is affected by the producer’s sensorimotor expectations about how they would act in the world, rather than by an implicit memory of how that action was actually produced.

In the current task, it is possible that participants were not judging the weight of the objects on the basis of attuning to the previous experience via memory at all. It might be that no such memory about weight was available, or, otherwise, explicable. Instead, a rational choice was made on the fly based on a heuristic that larger/smaller objects are heavier/lighter. It is unclear, however, why the use of such a strategy would be related to how speakers gesture about lifting the pieces. Instead, we argue that participants were most likely accessing a haptic knowledge for a simulation for what it felt like to lift the pieces when they made their weight judgments. Indeed, it has been found that, when judging an object’s weight, people actually imagine holding the object rather than using some kind of propositional rule (Klatzky, Lederman, & Matula, 1991). Furthermore, it has been found that previously experienced haptic information can be attuned to with some success, as haptic memory of objects even allows one to discover new properties of the object in imagery (Pouw, Aslanidou, Kamermans, & Paas, 2017; see also Kamermans et al., under review). Thus, although there is a possibility that participants only remembered visual properties of the objects and then used these visual properties to make heuristic inferences about the objects’ haptic properties, research suggests that this is not how people tend to judge haptic properties from memory.

We speculate that both gestures and weight estimates in the current context result from simulations that are neither the result of a “pure memory” of a previous action, nor the result of a rule-like “heuristic” that “small/large objects are lighter/heavier”. Rather, a simulation involves generation of information that abides by regularities observed in the real-world, and is not the simple replay of a previously performed action. This is based on the argument that actions are not represented by some motor plan that incorporates a list of muscle activations needed to perform the action successfully (Bernstein, 1966). Indeed, research (e.g., Kelso, Tuller, Vatikiotis-Bateson, & Fowler, 1984) has shown that when an action is initiated (e.g., speaking), perturbation of the trajectory of that action (e.g., locking the jaw in place) leads to fluid adjustments which recruit the other muscles than would, otherwise, be the case (e.g., lip adjustments), yielding a new sensorimotor solution to the same goal (e.g., speaking a syllable “|baeb|”). Thus, if gesture is derived from practical action, it is likely based on the broader ability to construct sensorimotor solutions constrained by contextual demands, rather than on memory of sensorimotor particulars. Of course, gestures are far less constrained by contextual demands than actions, because they do not involve real objects. This is precisely why gestures may reflect stereotypical sensorimotor solutions—gestures act out actions in ways that would fit most contexts (e.g., slower lifting gestures for heavier objects). A simulation is, therefore, a *constructive* process based on the accumulated sensorimotor knowledge that “smaller/larger objects are lighter/heavier”, but it goes beyond a simple heuristic, because it involves knowledge of real-world sensorimotor contingencies. We suggest that both producing gestures about interacting with the pieces and imagining the pieces so as to judge their weight rely on the simulation of expectations about how it would feel to interact with the pieces.

Importantly, however, in our study, we find no evidence that gesturing about the pieces in a certain way caused a change in how participants thought about the weight of the pieces. Experimentally manipulating gesture did not affect participants’ recollection or perception of the weights of the cubes. The fact that no significant differences between conditions were found and speech content did not affect perceived heaviness indicates that it is unlikely that our choice of conditions impacted our results. Instead, we observed an effect within the gesture condition—a correlation between how participants thought about the cubes and how they gestured about them. Participants’ utilization of two-handed gestures was related to the magnitude of the SWI they experienced, and the velocity with which they gestured was related to their recalled heaviness of the cubes. We speculate that, in both cases, how people thought about the task as they described it was reflected in gesture. Participants may have imagined the motor kinematics of the task to a greater or lesser degree as they were explaining the problem. Those more inclined to simulate the specifics of interacting with the cubes were more likely to produce two-handed gestures that showed the specific action required to manipulate the cubes, including the relative velocity of lifting light/heavy objects. Furthermore, participants who thought more specifically about the motor processes involved in the task as they were describing were also likely to form a weight judgment that was based on a *sensorimotor* judgment when asked to judge the objects’ weight. Such detailed sensorimotor imagination of the cubes (both during the description task and during the rating task) was particularly likely to be distorted by the sensorimotor know-how that large objects are generally heavy. Under this explanation, two-handed lifting gestures were an embodiment of detailed sensorimotor imagery about lifting, but were not a driving agent in whether participants adopted a detailed sensorimotor imagining or not.

An interesting aspect of this finding is how the judgments were biased when they were made from memory. In the classic SWI, participants judge a smaller cube that they are holding as heavier than a larger cube when the two are actually the same weight. Although we replicated this traditional SWI when the cubes were compared from immediate experience (the smaller cube was perceived as 43% heavier than the larger cube), when participants were asked to judge the heaviness of the cubes from memory, they seemed to judge heaviness rationally—that is, they reported that the larger cube had felt 21% heavier than the smaller cube.^4^ This effect was not predicted, and to our knowledge, this is the first study that has revealed an inverted SWI when weight judgments are made from memory (as the SWI is generally not studied in relation to the memory system). The previous research has indicated that people actually imagine holding an object when judging the object’s weight and other haptic properties (Klatzky et al., 1991), yet the observed inversion of the SWI in memory suggests that this active imagining is not operating with accurate implicit memories of how these particular objects were perceived as they were manipulated (which would have led to no illusion) or with accurate explicit memories of how these particular objects were perceived as they were manipulated (which would have led to the traditional smaller-is-heavier illusion). Instead, the judgment seems to be based on a stereotypical understanding that larger objects are typically heavier than smaller objects.

One of the reviewers of the current paper suggested that there is a possibility that the memory of sensorimotor experiences may be affected by sensorimotor expectations. Under this view, it is not so much that participants are drawing from a memory of a sensorimotor experience (e.g., small object being heavy), but rather from a transformed memory where unexpected aspects of the sensorimotor event (e.g., light objects feeling heavier) are “washed out”. The degree to which a memory is resolved in favor of a sensorimotor expectancy might then explain why gesture is related to heaviness ratings from memory. The task-relevant two-handed gesturing effectively is related to expectancy, not so much a memory. We are very favourable to this idea as we have argued that gestures are governed by a system that operates on a history of sensorimotor contingencies, rather than on the sensorimotor specifics of a moment ago, though the scope of the current results deems this idea still too speculative, and more research is needed to directly test this idea. Note that research on the SWI has confirmed that repeated interactions with objects can affect the illusion, suggesting that the sensorimotor history can be manipulated and is not some unchangeable pre-given. Namely, Flanagan, Bittner, and Johansson (2008) have found that after a multi-day training with interacting with objects that induce the SWI (240 lifts for 11 days), the SWI not only dissipates, it is inverted. Extensively trained participants judged larger objects as heavier than smaller objects that weighed the same. If gestures are indeed based on expectations that operate on slow timescales, we would expect that multi-day training with new sensorimotor contingencies would be (especially) apparent in the way that people gesture about such contingencies. Further research can explore our hypothesis that gestures attune to a history of sensorimotor interactions by assessing effects of multi-day training vs. immediate previous experiences when we gesture about object manipulations. Furthermore, perhaps, in the current paradigm, repeated sensorimotor imagining of the objects in gesture may come to affect sensorimotor expectations given enough time (Pouw & Hostetter, 2016).

To conclude, the present pre-registered study has offered novel insights into how gestures are related to memory of sensorimotor information. Although gesturing about the cubes did not affect participants’ memories of how heavy the objects were or their judgments of perceived heaviness when the objects were held again, the kinematics of gestures were related to speakers’ thinking about sensorimotor properties of the cubes. The results indicate that gestures reflect rather than affect the way that we think about and remember objects’ sensorimotor properties, at least in the context examined here. It appears that gestures do not just reveal information about the actions which a speaker performed; instead, they reveal how the speaker thinks about what they did.

## Appendix 1

See Table 1.

**Table 1:** Bivariate Pearson’s correlations among variables of the study

Measures	*M*	SD	1	2	3	4	5	6	7	8	9	10	11	12
1. Gestures (p/m)	15.91	4.15	–											
2. One-hand gestures (p/m)	2.39	2.70	0.247	–										
3. Two-hand gestures (p/m)	4.60	3.60	0.315*	− 0.618***	–									
4. Deictic gestures (p/m)	7.33	2.81	0.696***	0.192	− 0.168	–								
5. Words (p/m)	103.69	25.39	0.484***	0.234	− 0.033	0.411**	–							
6. Practice time (s)	234.36	80.50	0.113	− 0.240	0.112	0.123	− 0.160	–						
7. Explanation time (s)	126.09	7.77	− 0.053	0.161	− 0.146	− 0.020	0.088	0.030	–					
8. Small cube (recalled)	3.88	1.83	0.057	0.203	− 0.150	0.098	− 0.107	− 0.055	0.001	–				
9. Large cube (recalled)	4.71	1.58	0.416**	0.053	0.292*	0.142	0.155	0.002	− 0.174	0.218**	–			
10. SWI (recalled)	− 0.83	2.14	− 0.283*	0.139	− 0.368**	− 0.026	− 0.212*	− 0.049	0.130	0.694***	− 0.552***	–		
11. Small cube (perceived)	5.32	1.61	0.149	0.166	− 0.006	0.082	0.050	0.047	0.111	0.505***	0.489***	0.068	–	
12. Large cube (perceived)	3.72	1.37	0.158	0.061	0.041	0.204	0.079	− 0.000	0.016	0.543***	0.514***	0.082	0.485***	–
13. SWI (perceived)	1.60	1.53	0.005	0.120	− 0.048	− 0.115	− 0.020	0.050	0.101	0.042	0.052	− 0.002	0.617***	− 0.389***

*SWI* size-weight illusion (difference score of small–large cubes)

**p* < 0.05, ***p* < 0.01 *** *p* < 0.001

## Appendix 2

*Analysis 4: individual differences in practice time* We assessed whether differences in time spent practicing the task differed per condition. An ANOVA revealed that this was not the case (*F* < 1, BF’s_01_ = 6.82). Furthermore, there were small differences in the amount of time allotted (SD = 7.77 s) to explain the task, as one experimenter stopped the stopwatch when participants started to explain the rules of the task instead of the procedure of the task. This did not lead to significant differences in explanation time between the no-gesture vs. gesture condition, (*F* < 1, BF’s_01_ = 6.32). Neither practice time nor explanation time was statistically significant when added as covariates to the previous confirmatory analyses for the effect of Condition on heaviness ratings (*F*s < 1, BF’s_01_ > 3.46).

*Analysis 5: individual differences in speech and its relation with gesture* Although none of the participants in the explanation conditions (no-gesture and gesture condition) mentioned weight or heaviness during their explanations, nine participants mentioned size at least once. Of these, four were in the no-gesture condition, and five were in the gesture condition (mean number of mentions of size: no-gesture condition = 0.17, SD = 0.67, gesture condition = 0.45, SD = 1.61, *t*[104] = 1.18 *p* = .241, BF_01_ = 2.62, *d* = 0.23). In general, participants in the no-gesture condition spoke 97.00 (SD = 26.19) words per minute, as compared to 110.39 (SD = 22.90) words per minute for participants in the gesture condition. This difference in speech rate was statistically significant, *t*(104) = 2.80, *p* = .006, BF_10_ = 6.370, *d* = 0.54.

We further assessed relations between spoken words per minute, the number of mentions of weights and size, and gesture frequencies per minute (see also Table 1 in “Appendix 1”). First, there was a statistically significant positive correlation between number of words spoken and the total number of gestures (*p* < .001, BF_10_ = 120.135) and deictic gestures (*p* = .002, BF_10_ = 16.35). However, the number of words spoken did not significantly correlate with two-handed gestures (*p* = .176, BF_01_ = 5.69) or one-handed gestures (*p* = .264, BF_01_ = 1.45). No significant correlations were found for the number of mentions of size of the cubes and gesture frequencies.

Although the number of words spoken did not seem related to two-handed gestures, it did relate in a similar way to the inverted SWI. Namely, just as two-handed gestures were related to a larger inverted SWI from memory, higher speech rates also related to a larger inverted SWI, *β* = − 0.21, *t*(105) = − 2.21, *p* = .029, BF_10_ = 1.787. However, when both number of words and number of two-handed gestures per minute were entered in a single analysis (with participants from the gesture condition), only number of two-handed gestures was a significant predictor of the size of the illusion, *R*^2^ = 0.15, *F*(2, 25) = 4.33, *p* = .018; *β*_words_ = − 0.11, *t*(52) = − 0.86, *p* = .396; *β*_two-handed gestures_ = − 0.37, *t*(52) = − 2.84, *p* = .006. The predictive value of a model with only two-handed gestures as predictor (BF_10_ = 6.544) was better than a model with only speech (BF_10_ = 0.341) or with speech and two-handed gesture (BF_10_ = 2.840). Taken together, these results suggest that speech rate cannot account for the effect of two-handed gestures on recalled heaviness.

## Footnotes

1 This additional measure was added after realizing the experiment should include a material check (with the same participants) to see if the materials would induce a size-weight illusion under normal conditions (i.e., perceived heaviness while lifting two cubes of different sizes at the same time). At this point, 21 participants had already been tested and, therefore, we only have perceived heaviness ratings while holding the cubes from the remaining 138 participants.

2 Although these two-handed gestures were coded under different criteria as to isolate lifting movements, this measure significantly correlated (*r* = .73) with two-handed grasp/move gestures.

3 Note that BFs are always reported for the hypothesis that is most likely given the data. Therefore, BF_10_ (odds ratio for the alternative vs. null hypotheses given the data) provides evidence for the alternative hypothesis (supporting significant *p* values), whereas BF_01_ (odds ratio null/alternative) provides evidence for the null hypothesis (supporting non-significant *p* values). Thus, larger BFs always equal stronger evidence.

4 Note, however, that the inversion is not complete as it seems to be half the size of the original size-weight illusion, 21% versus 43% difference. Also, some participants did perceive the smaller object as heavier based on memory reducing the inverted size-weight illusion on the group level.
